# Hierarchical Si/ZnO trunk-branch nanostructure for photocurrent enhancement

**DOI:** 10.1186/1556-276X-9-469

**Published:** 2014-09-04

**Authors:** Chang Fu Dee, Su Kong Chong, Saadah Abdul Rahman, Fatin Saiha Omar, Nay Ming Huang, Burhanuddin Yeop Majlis, Muhamad Mat Salleh

**Affiliations:** 1 Institute of Microengineering and Nanoelectronics (IMEN), Universiti Kebangsaan Malaysia (UKM), Bangi 43600, Selangor, Malaysia; 2 Low Dimensional Materials Research Centre, Department of Physics, University of Malaya, 50603 Kuala Lumpur, Malaysia

**Keywords:** Hierarchical, Nanostructure, Zinc oxide, Silicon, Photocurrent

## Abstract

Hierarchical Si/ZnO trunk-branch nanostructures (NSs) have been synthesized by hot wire assisted chemical vapor deposition method for trunk Si nanowires (NWs) on indium tin oxide (ITO) substrate and followed by the vapor transport condensation (VTC) method for zinc oxide (ZnO) nanorods (NRs) which was laterally grown from each Si nanowires (NWs). A spin coating method has been used for zinc oxide (ZnO) seeding. This method is better compared with other group where they used sputtering method for the same process. The sputtering method only results in the growth of ZnO NRs on top of the Si trunk. Our method shows improvement by having the growth evenly distributed on the lateral sides and caps of the Si trunks, resulting in pine-leave-like NSs. Field emission scanning electron microscope image shows the hierarchical nanostructures resembling the shape of the leaves of pine trees. Single crystalline structure for the ZnO branch grown laterally from the crystalline Si trunk has been identified by using a lattice-resolved transmission electron microscope. A preliminary photoelectrochemical (PEC) cell testing has been setup to characterize the photocurrent of sole array of ZnO NR growth by both hydrothermal-grown (HTG) method and VTC method on ITO substrates. VTC-grown ZnO NRs showed greater photocurrent effect due to its better structural properties. The measured photocurrent was also compared with the array of hierarchical Si/ZnO trunk-branch NSs. The cell with the array of Si/ZnO trunk-branch NSs revealed four-fold magnitude enhancement in photocurrent density compared with the sole array of ZnO NRs obtain from VTC processes.

## Background

Homo- and hetero-hierarchical nanostructures (NSs) consist of two or more materials in the family of nanostructures have become one of the most intensively studied topics in the field of nanotechnology. Nanoparticles (NPs), nanowires (NWs) (including nanorods and nanowhiskers), nanolayers (NLs) (including nanoflakes and nanowalls), and other types of fundamental building blocks consist of a single material-NSs have been uncovered, synthesized, and studied for more than few decades ago. The next level of study based on hierarchical NSs is the combination/integration of more than one type of fundamental building blocks as mentioned above which may consist of more than one material. Many researchers' works for applications of hierarchical NSs actually show better performance compared with the primary building block NSs [1-3]. Those applications include hybrid nanoelectronic, nano-optoelectronic, nanomechanical, and electrochemical devices.

Recently, the characterization and implementation of hierarchical NSs in photoelectrochemical (PEC) cell has been widely explored [4,5]. Hierarchical core-shell or trunk-branch NSs are expected to give better performance to the photocurrent. Those are commonly addressed as photoconductors. A photoconductor is a device which will conduct electricity when exposed to light. Infrared detectors, optical imaging devices, photodetectors, photovoltaics, optical switches, biological and chemical sensing photocopiers, and optical receivers for fiber-optic communication all rely on the characteristic of a photoconductor. In the scale of nanometer, scientists believe that photoconductors will provide better answer for nanoelectronics, nano, and molecular scaled optical-related devices.

Basically, photocurrent could be sourced from two major mechanisms, namely photovoltaic and PEC processes. In photovoltaic process, photon from sun light generates free electron-hole pairs where they are then collected at the electrode, and electrical power could be extracted at the external circuit. For PEC process, absorbed photons are used to excite electrons and the excited electrons will drive the chemical reaction. One of the common examples for the second process is water splitting to generate hydrogen.

For visible light detection, Si as a group IV semiconductor material, is well-established due to its compatibility with CMOS process. It has been well-understood and studied. Up to date, some numbers of Si-based nanowires photoconductive devices have been studied [6-10]. Metal oxide NWs are also another important type of photosensitive materials. One of the most intensively studied materials is zinc oxide (ZnO) nanostructure. Its unique properties on magnetic, mechanical, optical, and the recent spintronics provide further opportunities on a wide variety of applications. Due to its wide bandgap (*E*_*g*_ = 3.37 eV at room temperature), applications as UV photodetector is possible. However, sparse literature showed photoresponse for a hierarchical NS consists both of Si and ZnO materials. In this work, hierarchical NS for a Si/ZnO trunk-branch array was fabricated and its initial photoactivity namely photocurrent was tested under one sun light irradiation.

## Methods

Crystal Si (111) (c-Si)- and indium tin oxide (ITO)-coated glass were used as substrates for ZnO deposition. Prior to the growth of ZnO nanorods (NRs), ZnO seed layers were spin-coated on the substrates. The colloidal solution was prepared by dissolving 0.2 M zinc acetate dehydrate and 0.2 M diethanolamine in ethanol and stirred at 60°C for 30 min. The solution was spin-coated onto the substrates at a spinning speed of 2,000 rpm for 30 s. The samples were then heated at 100°C for 15 min. The spin coating process was repeated three times. Subsequently, the samples were annealed at 300°C for 1 h in a Carbolite furnace to yield the ZnO seeds.

### Growth of ZnO NRs

ZnO nanorods were grown by two separate methods, namely hydrothermal growth (HTG) and vapor transport condensation (VTC) growth. Both growth processes have gone through the same seeding process as discussed above.

1. For HTG process. ZnO seeded substrates were placed into a beaker filled with mixture of 0.04 M Zn(NO_3_)_2_ and 0.04 M HMTA aqueous solution, and heated inside a laboratory oven at 90°C for 2 h. The as-grown ZnO NR samples were rinsed with deionized water for several times to remove impurities.

2. For VTC growth process. ZnO NRs were deposited onto the ZnO seeded substrates using a quartz tube furnace. Mixture of ZnO and graphite powder (ratio of 1:1) with a total weight of approximately 0.2 g was placed inside the center hot zone of the quartz tube. The added graphite powder was used to form eutectic for reducing the vaporized temperature of ZnO [11,12]. One end of the quartz tube was connected to N_2_ gas inlet, while the other end was remained open. The powder mixture was heated to 1,100°C for 1 h. The substrates were placed under a downstream of N_2_ flow, at about 12 cm from the powder boat. The substrate temperature was about 500°C at equilibrium.

### Synthesis of Si/ZnO trunk-branch NSs

**Figure 1:**
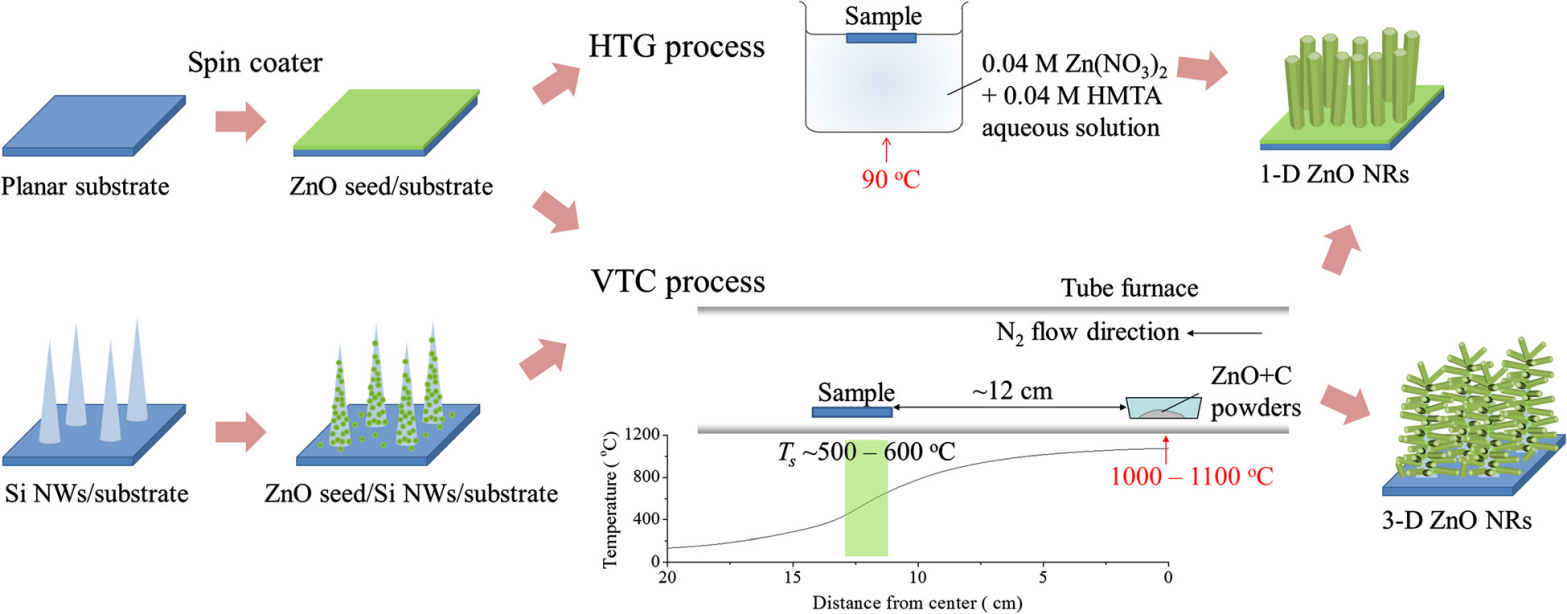
Schematic diagram describing the fabrication processes.

3-D Branching ZnO NRs were grown on a substrate pre-grown with Si NWs (Si NWs substrate) instead of new bare wafer. The Si NW arrays were synthesized by a plasma-assisted hot-wire chemical vapor deposition system using an indium catalyst [13-16]. Si NW array with average length and diameter of about 2 microns and 150 nm, respectively, acted as backbone (trunk) for the lateral growth of ZnO NRs. The similar ZnO seed layer preparation process was carried out on the Si NW substrate, and then it was followed by the deposition of ZnO NRs using VTC method. The synthesized processes for the ZnO NRs and Si/ZnO trunk-branch NSs are diagrammed and summarized in Figure 1.

### Characterization techniques

A Hitachi SU8000 field emission scanning electron microscopy (FESEM) was utilized for the morphological study of the samples. High-resolution transmission electron microscopy (HRTEM) micrographs of the samples were taken via a JEOL HRTEM (JEM-2100F), operating at an accelerating voltage of 200 kV. Characterization by X-ray diffraction and photoluminescence have been previously performed and published [17,18] (see Additional file 1). A preliminary PEC cell testing has been carried out to characterize the photocurrent. The prepared NSs on ITO-coated glass substrate were used as working electrode. The test was done by using a VersaSTAT 3 potentiostat (Ametek Princeton Applied Research, Oak Ridge, TN). A solar light simulator (Oriel Instrument) was used to generate an equivalent intensity of one sunlight (100 mWcm^−2^) AM 1.5 G radiation. A conventional three-electrode cell was constructed with the samples as working electrode, a platinum wire as counter electrode, and Ag/AgCl (in 3 M KCl) as reference electrode. The electrodes were immersed in a 1 M KCl electrolyte solution throughout the test. Since it was a PEC cell, the area of illumination is the same as the area which was immersed in the electrolyte, which was 1 cm × 2 cm^2^ for the sample of ZnO NRs as working electrode. While for Si/ZnO sample, it was 1 × 1 cm^2^. Current density was calculated in each case for comparison purpose.

## Results and discussion

**Figure 2:**
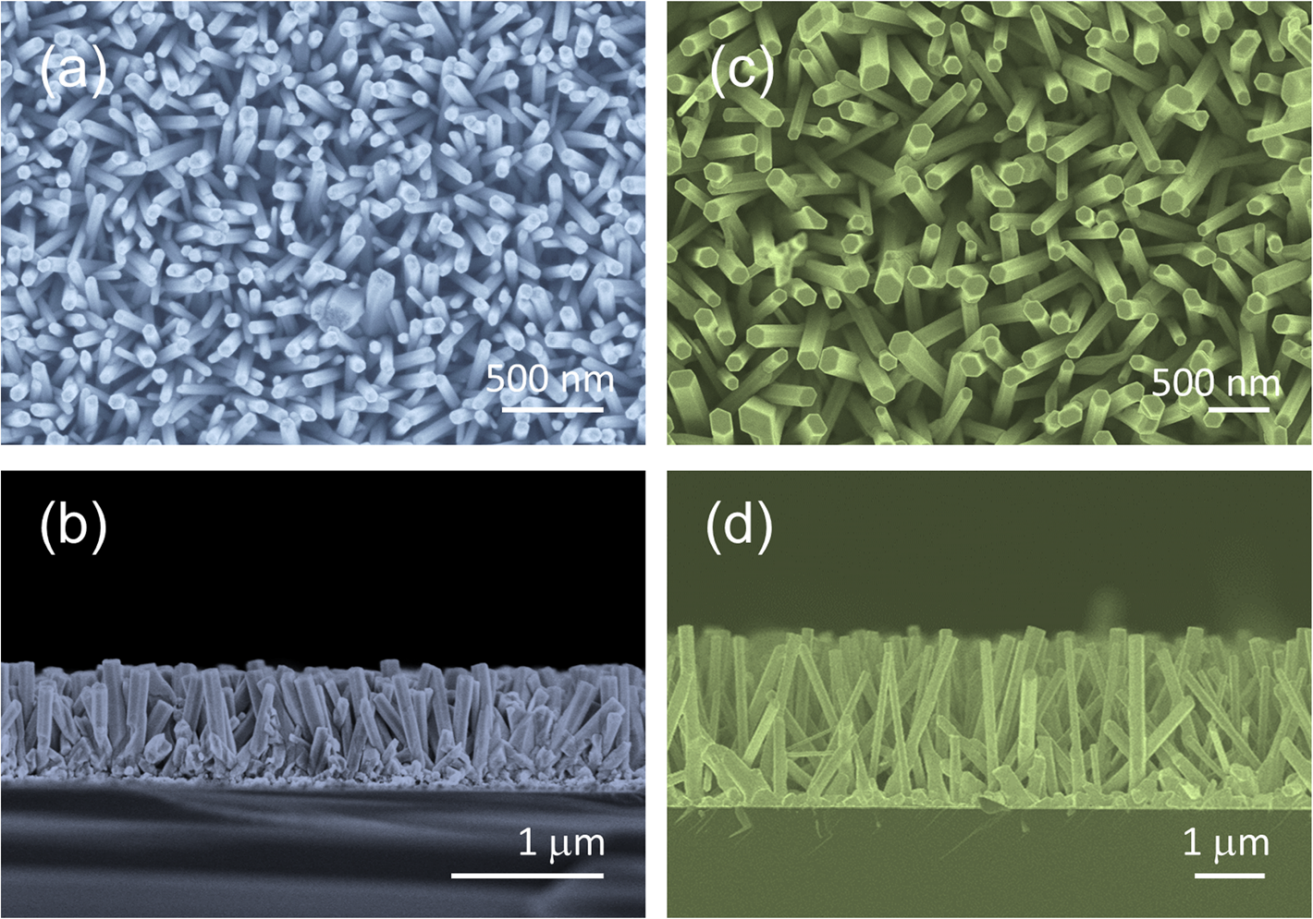
**Morphologies of the planar ZnO NRs.** Surface and cross-section FESEM images of the **(a, b)** HTG- and **(c, d)** VTC-grown ZnO NR arrays.

As shown by the FESEM images in Figure 2, both of the ZnO NRs grown by HTG and VTC methods show no difference in terms of general appearance. A well-defined hexagonal shape indicates crystalline structure of the ZnO NRs grown by both methods. But basically, the VTC-grown NRs are higher in diameters and lengths because the growth rate is higher for VTC method. Both of them show hexagonal structures while HTG-grown sample provides higher number of density.

**Figure 3:**
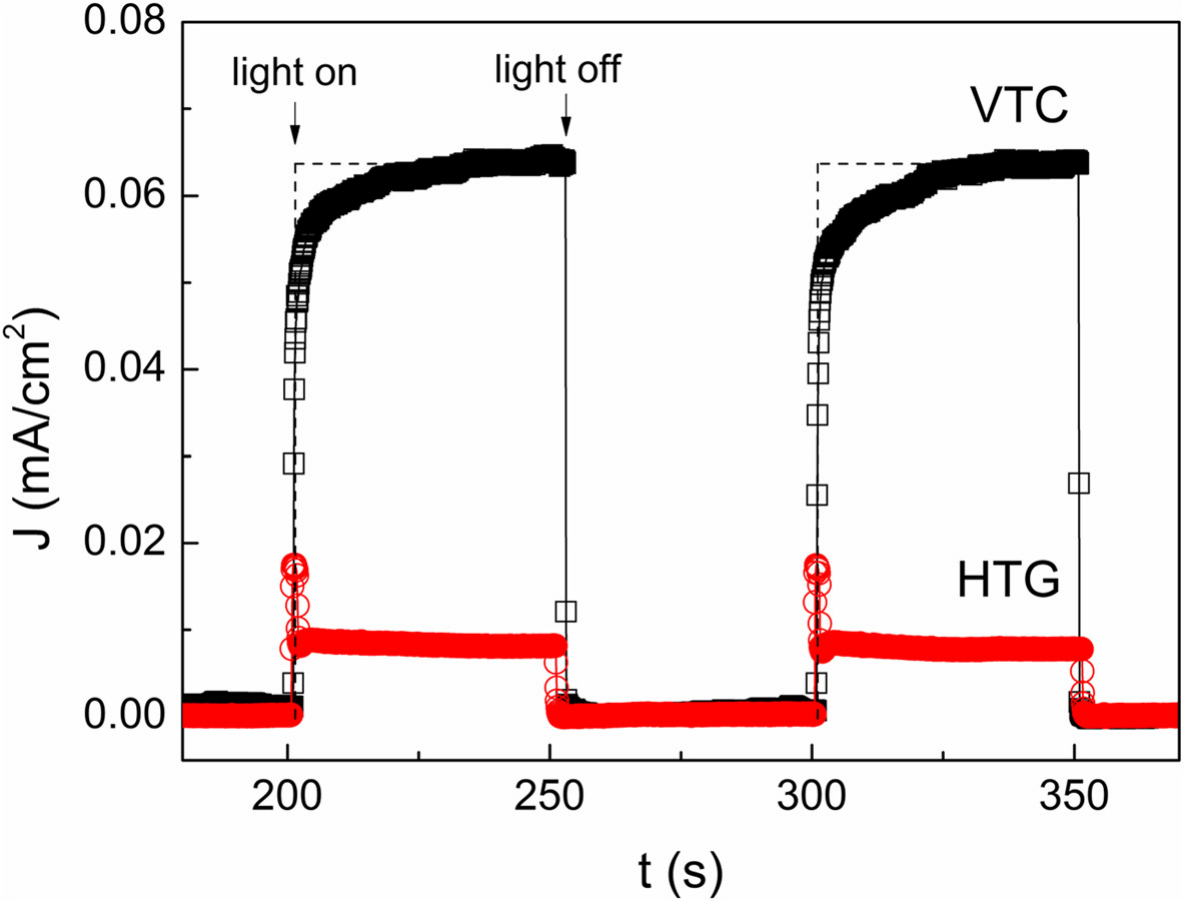
**Photocurrent of ZnO NRs.** Plot of photocurrent density (J) versus time (t) for one-dimensional ZnO NRs prepared by HTG and VTC methods.

Figure 3 shows the photocurrent-time plots of the as-grown ZnO NRs prepared on ITO-coated glass substrate using VTC and HTG methods. Despite of their similar morphologies, the VTC-grown ZnO NRs showed a higher significant photocurrent density (about 0.06 mA/cm^2^) compared to HTG-grown ZnO NRs (about 0.01 mA/cm^2^). Our results are comparable to the photocurrent density of the VTC-grown ZnO NWs (0.01 to 0.07 mA/cm^2^) [19] and HTG prepared-nitrogen-doped ZnO NRs (about 0.01 mA/cm^2^) [20] reported by other groups. The reason of the higher photocurrent effect for VTC-grown ZnO NRs could be due to the high temperature growth process, thus, resulted in the less structure defects in the ZnO NRs. However, the photocurrent response of the VTC-grown ZnO NRs was slower, which took more than 30 s for the current to reach its optimum value under illumination. The slow photocurrent response time for VTC-grown ZnO was also observed and reported separately by Dhara et al. [19] and Humayun et al. [21]. This can be due to the large quantity of absorbed oxygen created by the rapid photoresponse on the ZnO surface when illuminating by visible light, which slow down the photocurrent generation process [22].

**Figure 4:**
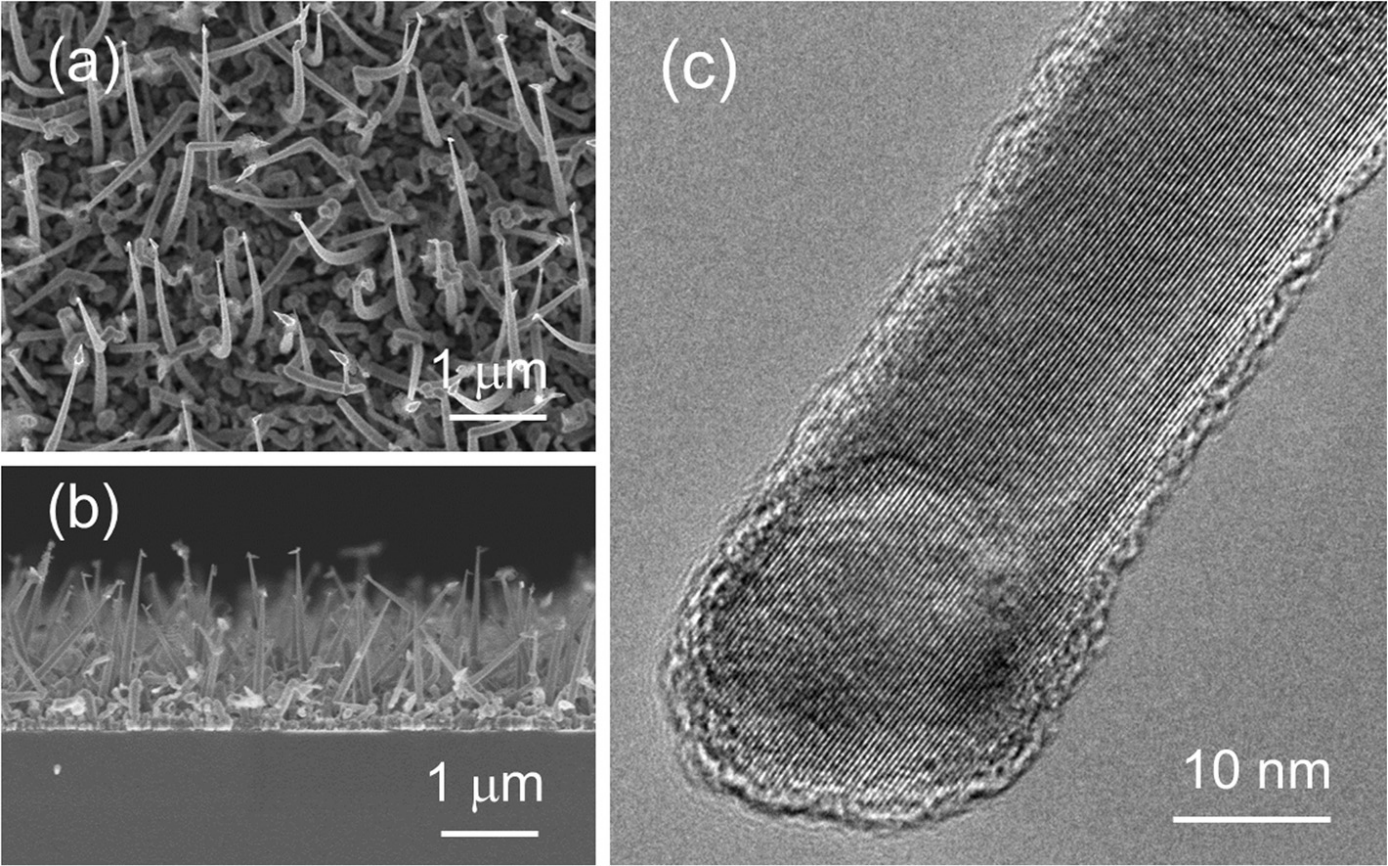
**Morphology of the Si NW trunk. (a)** Surface and **(b)** side morphologies of the Si NWs prepared by a plasma-assisted hot-wire chemical vapor deposition technique. **(c)** HRTEM micrograph of the Si NWs.

**Figure 5:**
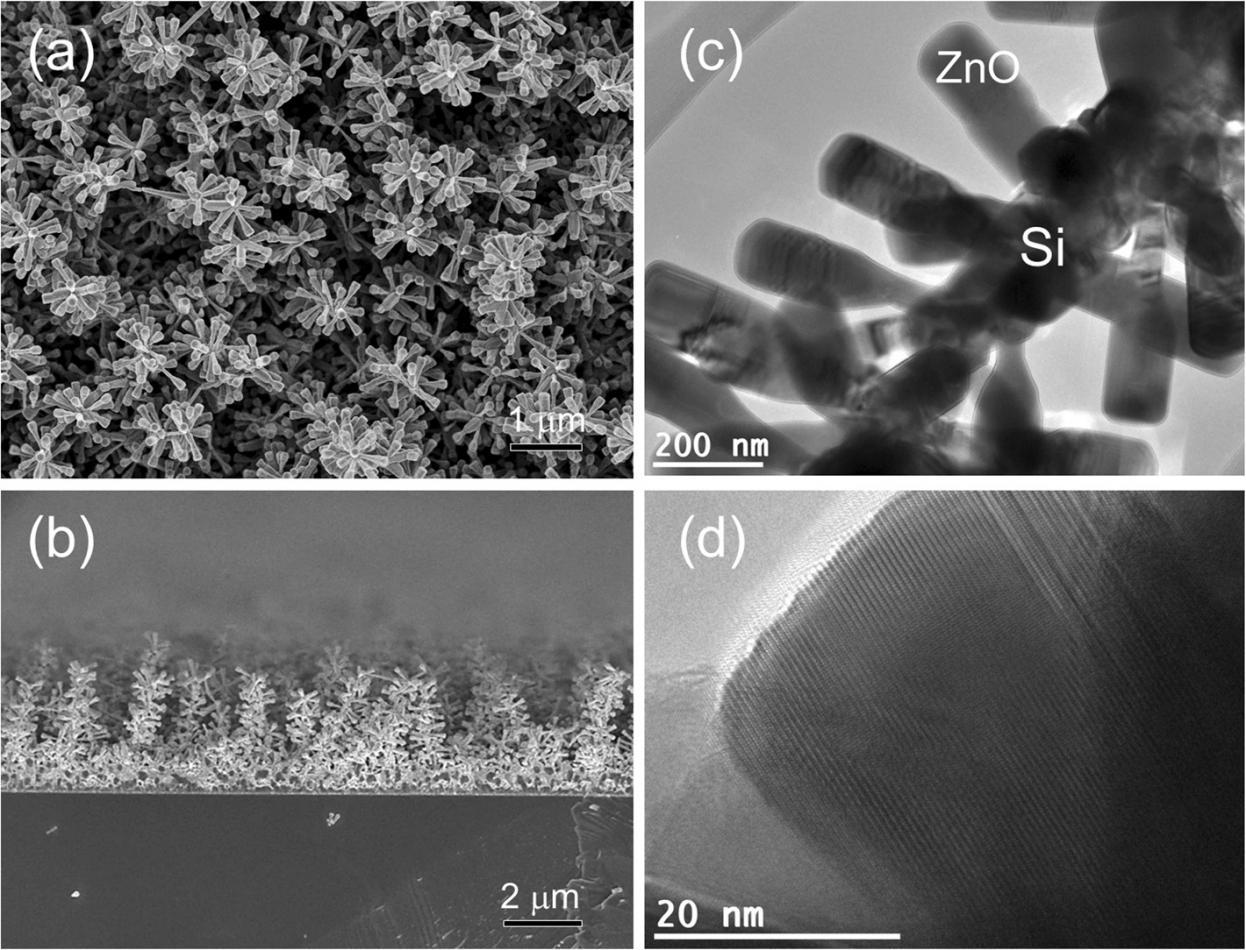
**3-D Si/ZnO hierarchical NWs.** FESEM **(a)** planar and **(b)** cross-section views of the Si/ZnO hierarchical NWs. **(c)** TEM image of a typical Si/ZnO hierarchical NW. **(d)** HRTEM micrograph taken from the ZnO branches.

In order to enhance the photoresponse of the VTC-grown ZnO NRs, the ZnO NRs were synthesized on the one-dimensional Si NWs trunk to induce the hierarchical Si/ZnO trunk-branch nanostructures for improvement in light trapping ability. Figure 4a,b shows the morphology of the Si NWs grown by our home-built plasma-assisted hot-wire chemical vapor deposition system. The length of the Si NWs is about 1 to 1.5 μm. HRTEM micrograph in Figure 4c shows that the NWs exhibit single crystalline structure. Note that the crystalline Si structure shows the greatest electrical conductivity, therefore, it serves as a good junction between ZnO NRs and the conducting electrode. The NWs reveal tapered morphology with base and top diameters of about 200 nm (Figure 4a,b) and 20 nm (Figure 4c), respectively. Basically, quantum effect of Si NWs will occur when the diameter of Si NW is less than 10 nm [23]. Therefore, it shows that Si NW will have the same bandgap as the bulk Si.FESEM images shown in Figure 5a,b are corresponded to the planar and side views of the hierarchical Si/ZnO trunk-branch NSs. It could be seen from the image that the lateral growth of ZnO NRs are evenly distributed on the sides and caps of the Si trunk nanowires. With the assistance of the ZnO seeds which acted as preferred growth sites, ZnO vapor molecules tend to absorb and elongate from the ZnO seeds on the surface of the Si NW trunk, forming ZnO NR branches. The size and distribution of the ZnO seeds on the Si NWs' surfaces thus play a crucial role in the growth of the Si/ZnO trunk-branch NSs. Estimation from the transmission electron microscope (TEM) image (Figure 5c) gives a length and diameter of about 300 and 120 nm, respectively, for the ZnO NR branches. In general, the length of the ZnO NR branches is much smaller than the VTC-grown planar ZnO NRs (nearly 2 μm) under the same deposition condition; however, the NRs' density per area is considerably higher. HRTEM micrograph in Figure 5d reveals an ordered lattice arrangement, indicating a single crystalline structure for the ZnO NR branches.

**Figure 6:**
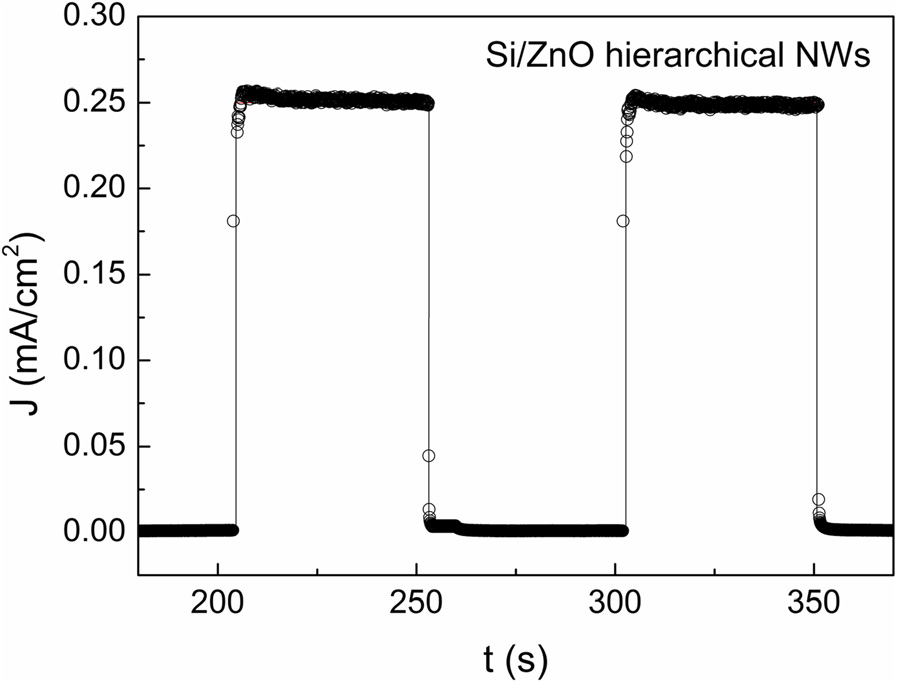
**Photocurrent of 3-D Si/ZnO hierarchical NWs.** Plot of photocurrent density (J) versus time (t) for the Si/ZnO hierarchical NWs prepared by VTC method.

In terms of photoactivity, Si trunk could act as a visible light sensitizer, and ZnO with wide bandgap of 3.37 eV could suppress the recombination of electron-hole pairs. With this combination, Si/ZnO trunk-branch NSs could absorb both visible light and UV light more effectively through different parts of the NSs, where the visible light and UV light would be absorbed at trunks and UV light at ZnO branches. For this hierarchical NS, photoelectric effect could be improved. The photocurrent density for hierarchical NSs where ZnO branches grown by VTC method shows significant improvement from 0.06 mA/cm^2^ (Figure 3) to 0.25 mA/cm^2^ (Figure 6). A design of alternating the on and off of the light was used to test the variation of photocurrents for two consecutive cycles. The Si/ZnO trunk-branch NSs show instant photocurrent response right after the light was switched on and it went straight to zero once the light was switched off. No residue current was found when the light was switched off. The whole response for the characterization process has been shown in Figure 6. In comparison with the VTC-grown planar ZnO NRs, the Si/ZnO trunk-branch NSs showed much shorter photocurrent response time (less than 2 s). We believed that the difference is due to the presence of Si trunk which improves the charge separation and mobility [24] and reduces the loss of photo-generated holes [25] in ZnO. As ZnO is transparent to visible light, the electron-hole pairs can also be created in the Si trunk. This facilitates the transportation of the photo-generated electron into the Si/ZnO interface, thus shorten the response time to reach optimum photocurrent. Additionally, the large potential barrier between the valence band of Si and ZnO [26] prevents the loss of photo-generated holes from recombination and contributes to the enhancement in the photocurrent.

As shown in Figure 6, under constant light radiation, the Si/ZnO trunk-branch NSs' photocurrent is gradually reducing over a period of 50 s within the measurement time. This may due to a less stability of the NSs. The same result was obtained for a similar hierarchical NS namely ZnO/Si broom-like nanowires by Kargar and co-workers [27]. The comparison is quiet relevant since both have the same materials and resemble the same structure. The only difference is that Kargar's NSs with the ZnO NRs is shown only on the top portion of the Si backbone NWs whereas our work shows NSs with ZnO NRs evenly distributed on the lateral side and cap of each Si trunk, although both researches show FESEM's images with quite similar number of density for Si trunk on the substrate and the similar HTG growth process for both our and Karger's experiments on the growth of ZnO NRs. Kargar's work produced broom-like nanowires whereas our work came out with the hierarchical nanostructures resembling the leaves of a pine tree. However, the seeding process for ZnO seeds was different. Kargar used sputtering process and we used spin coating method. The figure of merit by using spin coating process is the seeding could be evenly distributed in the whole lateral side of each Si trunk and resulted in the even growth of pine-leave-like NSs.

The discussion are extended to compare photocurrent effect of our Si/ZnO trunk-branch NSs with other popular photosensitive nanomaterials, for instance, TiO_2_[24,25] and InGaN [4]. Hwang et al. [25] synthesized high density Si/TiO_2_ core-shell NWs, and the photocurrent density is about 0.25 mA/cm^2^ under the illumination of 100 mWcm^−2^ full spectrum in a solar simulator, which has the same value as our Si/ZnO trunk-branch NSs. Our Si/ZnO trunk-branch NSs showed fairly higher photocurrent density compared to the Si/InGaN core-shell NW arrays (0.05 to 0.12 mA/cm^2^) demonstrated by Hwang et al. [4].

## Conclusions

An improved method has been used for the growth of Si/ZnO trunk-branch NSs where the ZnO NRs could be distributed more evenly on the lateral side and cap of each Si trunk. The photocurrent of the NSs have been measured and compared to the sole ZnO NRs. Significant improvement was recorded for this hierarchical Si/ZnO NS array.

## Competing interests

The authors declare that they have no competing interests.

## Authors’ contributions

CF drafted the manuscript, amended the final version, and contributed to the explanation and analysis of the data. SA and SK conceived the study. SK participated in the experiment, performed most of the samples' characterizations. SA also provided the solutions and support on multiple problems for the growth of Si NWs and analysis of the materials. FS and MN participated in the design of the photocurrent measurement and analysis. BY and MM provided opinions on some problems. All authors read and approved the final manuscript.

## Supplementary Material

Additional file 1 Supplementary data for hierarchical Si/ZnO trunk-branch nanostructure for photocurrent enhancement. Click here for file

## References

[B1] Gao P-X Shimpi P Gao H Liu C Guo Y Cai W Liao K-T Wrobel G Zhang Z Ren Z Lin H-J Hierarchical assembly of multifunctional oxide-based composite nanostructures for energy and environmental applications Int J Mol Sci 2012 9 6 7393 7423 22837702 10.3390/ijms13067393 PMC3397534

[B2] Alenezi MR Henley SJ Emerson NG Silva SRP From 1D and 2D ZnO nanostructures to 3D hierarchical structures with enhanced gas sensing properties Nanoscale 2014 9 235 247 10.1039/c3nr04519f 24186303

[B3] Lee J-H Gas sensors using hierarchical and hollow oxide nanostructures: overview Sensors Actuators B 2009 9 319 336 10.1016/j.snb.2009.04.026

[B4] Hwang YJ Wu CH Hahn C Jeong HE Yang P Si/InGaN core/shell hierarchical nanowire arrays and their photoelectrochemical properties Nano Lett 2012 9 3 1678 1682 10.1021/nl3001138 22369381

[B5] Kim H Yong K Highly efficient photoelectrochemical hydrogen generation using a quantum dot coupled hierarchical ZnO nanowires array ACS Appl Mater Interfaces 2013 9 24 13258 13264 10.1021/am404259y 24274430

[B6] Ahn Y Dunning J Park J Scanning photocurrent imaging and electronic band studies in silicon nanowire field effect transistors Nano Lett 2005 9 1367 1370 10.1021/nl050631x 16178240

[B7] Servati P Colli A Hofmann S Fu YQ Beecher P Durrani ZAK Ferrari AC Flewitt AJ Robertson J Milne WI Scalable silicon nanowire photodetectors Physica E: Low-dimensional Systems and Nanostructures 2007 9 64 66 10.1016/j.physe.2006.12.054

[B8] Kim KH Keem K Jeong DY Min BD Cho KA Kim H Moon B Noh T Park J Suh M Kim S Photocurrent of undoped, n- and p-type Si nanowires synthesized by thermal chemical vapor deposition Jpn J Appl Phys 2006 9 4265 4269 Part 1 10.1143/JJAP.45.4265

[B9] Choi HG Choi YS Jo YC Kim H A low-power silicon-on-insulator photodetector with a nanometer-scale wire for highly integrated circuit Jpn J Appl Phys 2004 9 3916 3918 Part 1 10.1143/JJAP.43.3916

[B10] Park J-H Kim H Wang I-S Shin J-K Quantum-wired MOSFET photodetector fabricated by conventional photolithography on SOI substrate4th IEEE Conference on Nanotechnology (NANO-04) 2004 Munich, Germany: IEEE New York 425 427

[B11] Fu DC Majlis BY Yahaya M Salleh MM Electrical characterization of cross-linked ZnO nanostructures grown on Si and Si/SiO_2_ substrate Sains Malays 2008 9 3 281 283

[B12] Karamdel J Dee CF Saw KG Varghese B Sow CH Ahmad I Majlis BY Synthesis and characterization of well-aligned catalyst-free phosphorus-doped ZnO nanowires J Alloys Compd 2012 9 68 72 10.1016/j.jallcom.2011.09.018

[B13] Chong SK Dee CF Yahya N Rahman SA Control growth of silicon nanocolumns’ epitaxy on silicon nanowires J Nanopart Res 2013 9 1571

[B14] Chong SK Goh BT Apanut Z Muhamad MR Dee CF Rahman SA Synthesis of indium-catalyzed Si nanowires by hot-wire chemical vapor deposition Mater Lett 2011 9 2452 2454 10.1016/j.matlet.2011.04.100

[B15] Chong SK Goh BT Dee CF Rahman SA Effect of substrate to filament distance on formation and photoluminescence properties of indium catalyzed silicon nanowires using hot-wire chemical vapor deposition Thin Solid Films 2013 9 153 158

[B16] Chong SK Goh BT Dee CF Rahman SA Study on the role of filament temperature on growth of indium-catalyzed silicon nanowires by the hot-wire chemical vapor deposition technique Mater Chem Phys 2012 9 635 643 10.1016/j.matchemphys.2012.05.037

[B17] Chong SK Dee CF Rahman SA Structural and photoluminescence investigation on catalytic growth of Si/ZnO heterostructure nanowires Nanoscale Res Lett 2013 9 174 10.1186/1556-276X-8-174 23590803 PMC3637626

[B18] Chong SK Lim EL Yap CC Chiu WS Dee CF Rahman SA Hierarchical-oriented Si/ZnO heterostructured nanowires Sci Adv Mater 2014 9 782 792 10.1166/sam.2014.1768

[B19] Dhara S Giri PK Enhanced UV photosensitivity from rapid thermal annealed vertically aligned ZnO nanowires Nanoscale Res Lett 2011 9 504 10.1186/1556-276X-6-504 21859456 PMC3212019

[B20] Game O Singh U Gupta AA Suryawanshi A Banpurkar A Ogale S Concurrent synthetic control of dopant (nitrogen) and defect complexes to realize broadband (UV–650 nm) absorption in ZnO nanorods for superior photo-electrochemical performance J Mater Chem 2012 9 17302 17310 10.1039/c2jm32812g

[B21] Humayun Q Kashif M Hashim U Qurashi A Selective growth of ZnO nanorods on microgap electrodes and their applications in UV sensors Nanoscale Res Lett 2014 9 29 10.1186/1556-276X-9-29 24423232 PMC3912502

[B22] Kao CY Hsin CL Huang CW Yu SY Wang CW Yeh PH Wu WW High-yield synthesis of ZnO nanowire arrays and their opto-electrical properties Nanoscale 2012 9 1476 10.1039/c1nr10742a 22012241

[B23] Ma DDD Lee CS Au FCK Tong SY Lee ST Small-diameter silicon nanowire surfaces Science 2003 9 1874 1877 10.1126/science.1080313 12595610

[B24] Devarapalli RR Debgupta J Pillai VK Shelke MV C@SiNW/TiO_2_ core-shell nanoarrays with sandwiched carbon passivation layer as high efficiency photoelectrode for water splitting Scientific Reports 2014 9 4897 24810865 10.1038/srep04897 PMC4014881

[B25] Hwang YJ Boukai A Yang P High density n-Si/n-TiO_2_ core/shell nanowire arrays with enhanced photoactivity Nano Lett 2009 9 1 410 415 10.1021/nl8032763 19053790

[B26] Um HD Moiz SA Park KT Jung JY Jee SW Ahn CH Kim DC Cho HK Kim DW Lee JH Highly selective spectral response with enhanced responsivity of n-ZnO/p-Si radial heterojunction nanowire photodiodes Appl Phys Lett 2011 9 3 033102 10.1063/1.3543845

[B27] Kargar A Sun K Kim SJ Lu D Jing Y Liu Z Pan X Wang D Three-dimensional ZnO/Si broom-like nanowire heterostructures as photoelectrochemical anodes for solar energy conversion Phys Status Solidi A 2013 9 12 2561 2568 10.1002/pssa.201329214

