# Supplementary material for: Hierarchical Si/ZnO trunk-branch nanostructure for photocurrent enhancement
Source: Nanoscale Res Lett. 2014 Sep 4;9(1):469. doi: 10.1186/1556-276X-9-469 (PMC4160322; doi:10.1186/1556-276X-9-469)

**Supplementary Data for**

**Hierarchical Si/ZnO trunk-branch nanostructure for photocurrent enhancement**

Chang Fu Dee^1^*, Su Kong Chong^2^, Saadah Abdul Rahman^2^, Fatin Saiha Omar^2^, Nay Ming Huang^2^, Burhanuddin Yeop Majlis^1^, Muhamad Mat Salleh^1^

^1^Institute of Microengineering and Nanoelectronics (IMEN), Universiti Kebangsaan Malaysia (UKM), Bangi, Selangor, Malaysia.

^2^Low Dimensional Materials Research Centre, Department of Physics, University of Malaya, 50603 Kuala Lumpur, Malaysia.

* Corresponding author

Email: [deechangfu@gmail.com](mailto:deechangfu@gmail.com)

XRD patterns of the Si NWs and Si/ZnO hierarchical NWs.


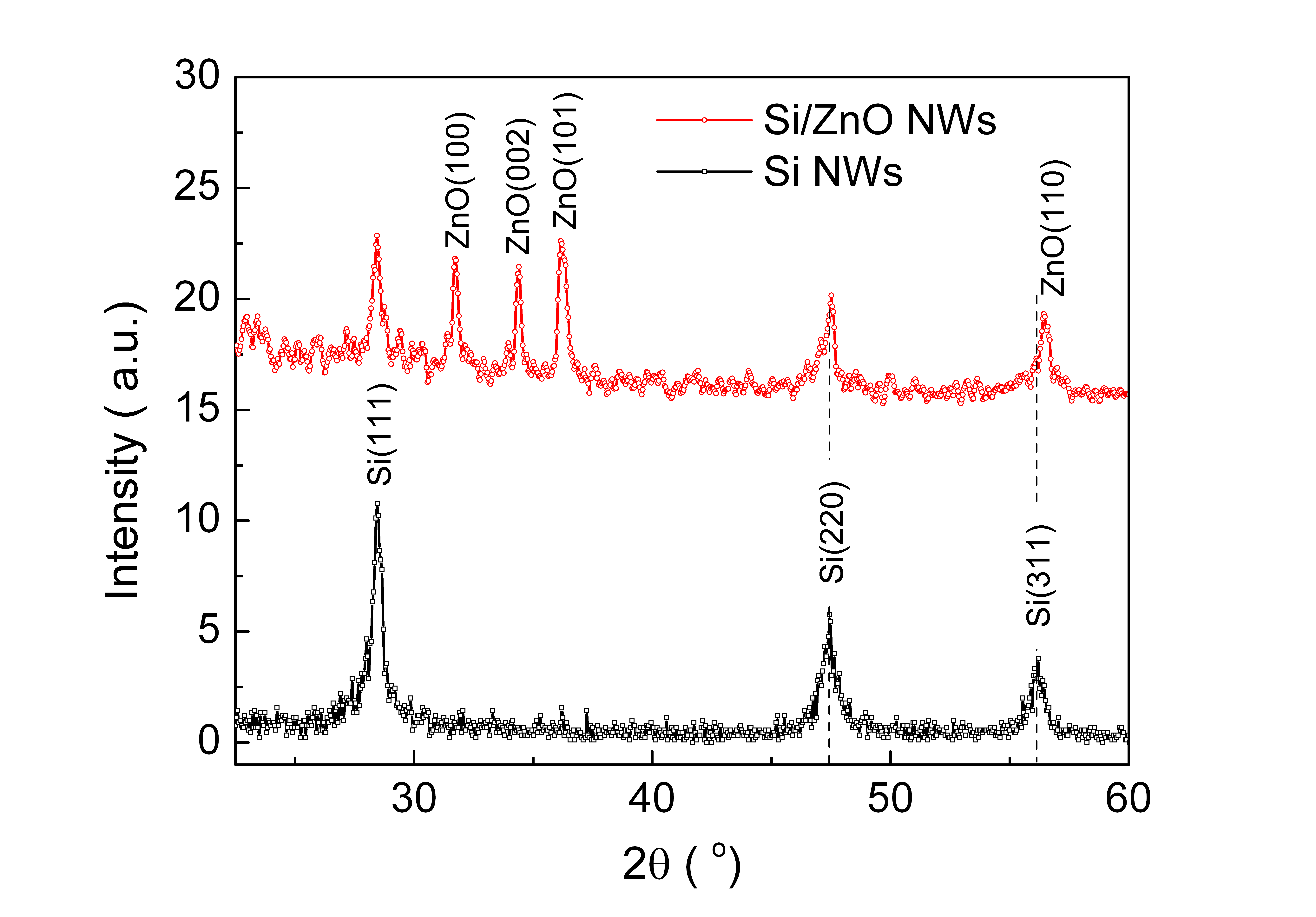


PL spectra of the Si NWs, ZnO NRs and Si/ZnO hierarchical NWs obtained at an excitation wavelength of 325 nm.


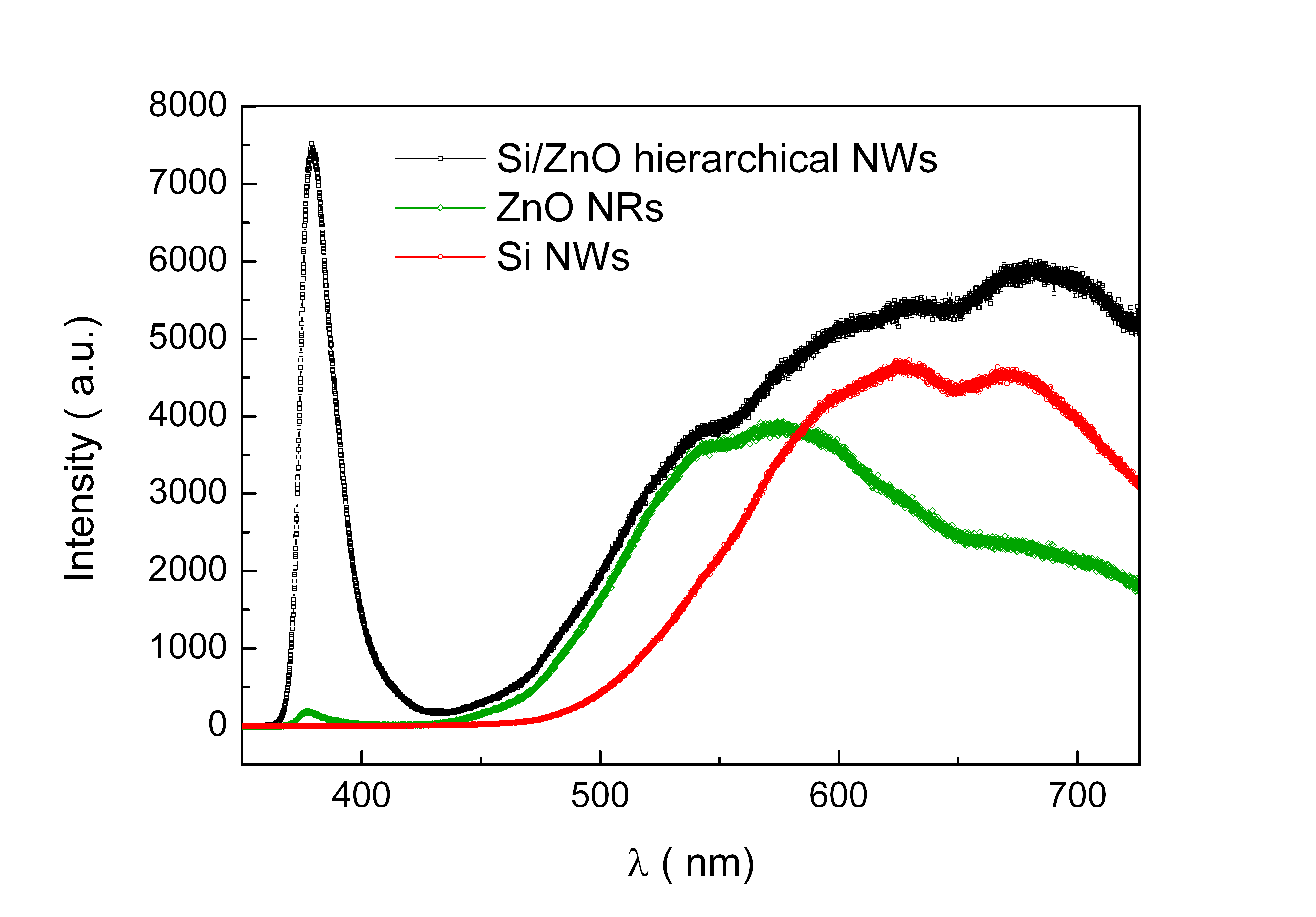

Supplement: Additional file 1 — Supplementary data for hierarchical Si/ZnO trunk-branch nanostructure for photocurrent enhancement. [file 1556-276X-9-469-S1.docx]
